# Systematic review and meta-analysis of iodine deficiency and its associated factors among pregnant women in Ethiopia

**DOI:** 10.1186/s12884-021-03584-0

**Published:** 2021-02-04

**Authors:** Robel Hussen kabthymer, Mohammed Feyisso Shaka, Getnet Melaku Ayele, Bereket Geze malako

**Affiliations:** 1 College of Health sciences and Medicine, School of Public Health, Dilla University, P.O. Box 419, Dilla, Ethiopia; 2 College of Health sciences and Medicine, Department of Midwifery, Dilla University, P.O. Box 419, Dilla, Ethiopia; 3 World Vision Ethiopia, Sodo Program coordination office, Wolaita Sodo, Ethiopia

**Keywords:** Urine iodine, Deficiency, Prevalence, Magnitude, Goiter, Micronutrient

## Abstract

**Background:**

Iodine deficiency (ID) is a global public health problem and its impact is more pronounced in low-income countries. During pregnancy, iodine requirement is known to elevate sharply, making pregnant women, especially those living in low-income countries highly vulnerable to iodine deficiency. This study aims to assess the prevalence of iodine deficiency and its associated factors among pregnant women in Ethiopia.

**Methods:**

A systematic literature search was performed by using PubMed, CINAHL, Web of science, global health, and Google scholar electronic databases. Two authors independently extracted all the necessary data using a structured data extraction format. Data analysis was done using STATA Version 14. The heterogeneity of the studies was assessed by using I^2^ test. A random-effects model was used to estimate the pooled prevalence and pooled odds ratio. The presence of publication bias was checked using Funnel plot and Egger’s test.

**Results:**

One thousand one hundred and sixteen studies were reviewed and seven studies fulfilling the inclusion criteria were included in the meta-analysis. The meta-analysis of seven studies that included 2190 pregnant women showed a pooled prevalence of iodine deficiency during pregnancy to be 68.76% (95% CI: 55.21–82.31). In a subgroup analysis, the prevalence in Oromia region is 71.93% (95% CI: 54.87–88.99) and in Amhara region is 60.93% (95% CI: 57.39–64.48). Iodized salt use (AOR = 0.18; 95% CI: 0.08–0.44) and 1st trimester pregnancy (AOR = 0.68; 95% CI: 0.47–0.99) were found to have a significant association with iodine deficiency.

**Conclusions:**

The prevalence of iodine deficiency during pregnancy using urine iodine is considerably high in Ethiopia. Using iodized salt is found to reduce the burden. Hence, there is a need to strengthen iodization programs to tackle the problem.

**Supplementary Information:**

The online version contains supplementary material available at 10.1186/s12884-021-03584-0.

## Background

Iodine is an indispensable micronutrient and essential component of the thyroid hormones thyroxine (T4) and triiodothyronine (T3), which are vital for normal growth and metabolism during pregnancy, infancy, and throughout life [1]. Iodine deficiency (ID) is a public health problem globally. Although iodine deficiency affects both developed and developing countries, the impact is more frequently observed in low-income countries [2].

In 2011, globally an estimated 1.8 billion people had inadequate amounts of iodine in their diet and are at risk of iodine deficiency disease (IDD) [3]. Iodine deficiency is the world’s most prevalent, yet easily preventable, cause of brain damage [4]. In 2011, Ethiopia was listed among the most iodinedeficient countries in the world [5]. The magnitude of goiter in Ethiopia ranges from 5 to 60%, implying, a mild to severe IDD degree [2].

The amount of iodine needed by our body varies by different factors like physiological changes, including pregnancy. The iodine requirement during pregnancy is known to increase sharply because i) there is a 50% increase in maternal thyroxine (T4) production needed to maintain maternal euthyroidism and meet the thyroid hormone requirements of the fetus; ii) iodine needs to be transferred to the fetus for fetal production of thyroid hormone, specifically in later gestation stages; and iii) probable increment in renal iodine clearance. Adequate iodine intake is essential during pregnancy for fetal and maternal production of thyroid hormones which is critical for optimal fetal neurodevelopment [6].

Inadequate iodine in our diet leads to a range of poor health outcomes. The consequences of severe iodine deficiency (ID) during pregnancy include spontaneous abortion, stillbirth, congenital abnormalities, and endemic cretinism [2]. Mounting evidence suggests that even transient mild gestational ID has subtle negative impacts including reductions in IQ [6] and poor educational outcomes [7].

Recently WHO/UNICEF increased the recommended nutrient intake (RNI) for iodine during pregnancy from 200 to 250 μg/d. Inadequate iodine intake below the recommended level in children and women can be assessed using urine iodine concentration (UIC) and thyroid volume (goiter rate) (GR) [4]. The recommended method to assess pregnant women’s iodine level is urinary iodine concentration (UIC) [2]. Because > 90% of dietary iodine eventually appears in the urine, UIC is an excellent indicator of recent iodine intake [1] but no published review incorporates reports from articles that used urine iodine to assess iodine deficiency in Ethiopia.

As one of the bold strategies to combat the highly prevalent iodine deficiency, in 2011, Ethiopia’s government passed comprehensive legislation that mandates the use of iodized salt for human consumption [8]. WHO recommends that the coverage of iodized salt utilization at the household level should be above 90% to eliminate iodine deficiency in countries with salt iodization programs [9]. In Ethiopia, the household level coverage of iodized salt has increased significantly over the years, from 15% in 2011 to 89% in 2016 [10]. Nevertheless, a national survey completed in 2014 by Ethiopian Public Health Institute (EPHI) reported that the iodine level in table salt was adequate only in 53.9% of the households [8].

Despite the government’s implementation of a long-term plan to eradicate ID through salt iodization, the problem reportedly persists. Moreover, evidence concerning vulnerable groups like pregnant women is incomplete. Therefore, we conducted this systematic review and Meta-analysis to assess the prevalence of iodine deficiency and its associated factors among pregnant women in Ethiopia.

## Methods

### Searches

A systematic literature search was performed by using PubMed, Cochrane library, CINAHL, and Google scholar. We applied Boolean operator like “AND”, “NOT” and “OR”. Through consideration of the Boolean operator we searched as follows: ((“Iodine OR Iodine”[Mesh]) AND (level OR Status OR deficiency [Subheading]) AND (Pregnant Women [Mesh]) AND “Ethiopia”[Mesh])) (Supplemental file 1)

### Eligibility criteria

Based on the mentioned inclusion and exclusion criteria, abstracts were reviewed from search results.

### Inclusion criteria

Study area: studies conducted in Ethiopia only.

Study design: observational studies (cross-sectional, case-control, and cohort studies).

Language: only studies published/written in the English language.

Population: studies conducted among pregnant women.

Publication issue: both published and unpublished articles were searched.

Study period: 2010–2020.

Context: Researches that have been conducted in Ethiopia using urine iodine concentration (UIC) to assess iodine deficiency were only included.

### Types of studies included

Systematic searching of the studies was undertaken from the 10th of March, 2020 to the 15th of May, 2020. All results were limited to articles published in English Language from 2010 till May 2020 G.C. Additionally all observational studies (case-control, cross-sectional, and cohort) were included. Case reports and case series were excluded from this study.

Initially, availability of full text titles and abstracts of the articles were assessed. Then the full papers of relevant articles were reviewed. Articles with inaccessible full papers and those published before 2010 were excluded from the analysis.

### Data extraction

Two authors (RH and MF) independently extracted all the necessary data using a standardized data extraction format prepared in Microsoft Excel. Disagreements between the authors during data extractions were discussed and reached on consensus. The data extraction format includes the name of the first author, publication year, name of the region, number of samples, response rate, median UIC, the method used to measure UIC, and prevalence with 95% CI. For the second outcome (associated factors), data were extracted in a two by two table format and then the odds ratio for each factor was calculated based on the findings of the original studies.

### Outcome measurement

There are two main outcomes in this study. The prevalence of iodine deficiency was estimated by dividing the total number of iodine-deficient cases by the total number of pregnant women participating in the studies then multiplying by 100. The second outcome was associated factors of iodine deficiency in pregnant women. For major determinants, the odds ratio was calculated based on binary outcomes from the primary studies. The factors included in this review were: use of iodized salt (Yes versus No), educational status of women (unable to read and write versus able to read and write), and trimester/stage of pregnancy (1st versus 2nd, 1st versus 3rd and 2nd versus 3rd).

### Risk of bias

For assessing the quality of the studies, Newcastle-Ottawa Scale (NOS) quality assessment tool was used [11]. The tool has indicators consisting of three main parts in which, the first part has five components and it assesses the methodological quality of each study. The second part of the assessment tool examines the comparability of the studies. The last part of the tool measures the quality of the original articles for their statistical analysis. Using the assessment tool, two authors independently evaluated the qualities of the original articles. The quality of each study was evaluated using these parameters. Those with medium quality (fulfilling 50% of quality assessment criteria) and high quality (> = 6 out of 10 scales) were included for analysis. Disagreements between assessors were solved by taking the mean score of their assessment results.

### Data processing and analysis

After extraction, the data were imported to STATA Version 14.0 statistical software for analysis. Heterogeneity among the reported prevalence was assessed by using I^2^ test static and its *p*-value [12]. As the test statistic showed, there is significant heterogeneity among the studies (I ^2^ = 98.3%, *p* = 0.000). As a result, random effect meta-analysis model was used to estimate the pooled prevalence. To reduce the random effect variations between the point estimates of the primary studies, a subgroup analysis was done based on the study region (Oromia and Amhara) where the study was conducted and study sample size. Moreover, univariate Meta-regression was also done to identify the possible source of heterogeneity by considering the year of publication, the region of the country, and sample size. Furthermore, we conducted influential sensitivity (leave one out) analysis to examine the effect of individual study on the observed heterogeneity.

Egger’s test at a 5% significance level was used to assess publication bias [13]. Pooled prevalence with 95% confidence intervals was presented using a forest plot format. For the second outcome, the odds ratio was used to determine the association between determinant factors and iodine deficiency among pregnant women in the included studies. A pooled odds ratio with 95% confidence intervals was presented using forest plot format. For reporting the findings, the ‘Preferred Reporting Items for Systematic Reviews and Meta-Analyses (PRISMA)’ protocol was used.

## Results

### General characteristics of studies

**Fig. 1:**
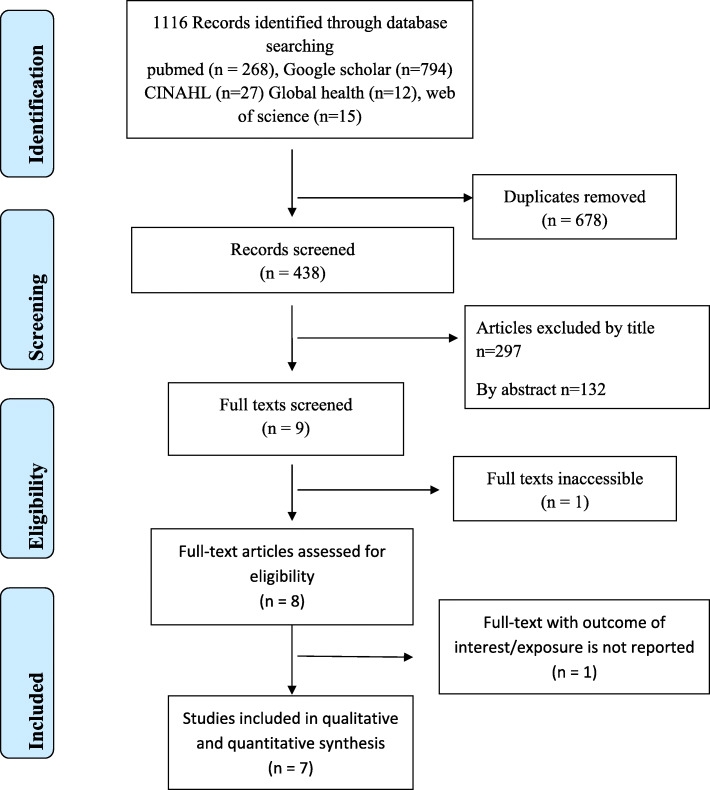
PRISMA flow diagram showing data extraction process for systematic review and meta-analysis of the prevalence and associated factors of iodine deficiency among pregnant women in Ethiopia, 2020

As shown in Fig. 1 below, in the first screening step we got a total number of 1116 papers but 678 papers were excluded due to duplication. Then, after assessing the titles and abstract, 429 papers were excluded. Nine papers were found suitable but the full text of one paper was inaccessible. After assessing the full text of 8 papers, 1 paper was excluded. Finally, 7 studies were included for analysis.

### Description of included studies

A total of 2190 pregnant women were recruited and involved in the included seven studies. The smallest and largest number of participants included in a single study was 40 and 435 respectively. All the included studies were cross-sectional studies and were published between 2010 and 2020. See Table 1.

**Table 1:** Summary of 7 studies included in the systematic review and meta-analysis of the prevalence and associated factors of iodine deficiency among pregnant women’s in Ethiopia, 2020

No	Author	Year	Region	Study area	Sample size	Response rate	Quality score	Median UIC	Method of UIC	Prevalence with 95% CI
1	Mengistu et al. [14]	2018	Oromia	Ada	356	100	10	85.7	Mass Spectrometry	77.6 (73.0–82.0)
2.	Wubet et al. [15]	2018	Amhara	Gondar	378	100	9	137	Spectrophotometer	60.5 (55–65.5)
3	Haji et al. [16]	2014	Oromia	Haramaya	435	82.8	10	58.1	Spectrophotometer	82.7 (79.1–86.5)
4	Zenebe et al. [17]	2014	Oromia	Jimma	423	100	9	48	spectrophotometer	88.9 (85.8–92.0)
5.	Dereje&Jemal [18]	2013	Amhara	Lai-gayint	350	100	9	110	spectrophotometer	61.4 (56.3–66.6)
6	Zeineba N [19]	2016	Oromia	Bishoftu	208	100	9	194.1	spectrophotometer	30.3 (24.0–36.6)
7.	Keno et al. [20]	2017	Oromia	Aira	40	100	8	88.6	spectrophotometer	80 (67.6–92.4)

### Risk of bias

The risk of bias for each original study was assessed by using Newcastle-Ottawa assessment tool which encompassed 10 different items (31). Among the 7 articles included, our summary assessment shows that all (100%) of the included studies had a low risk of bias. (Supplemental file 2).

### Prevalence of iodine deficiency

**Fig. 2:**
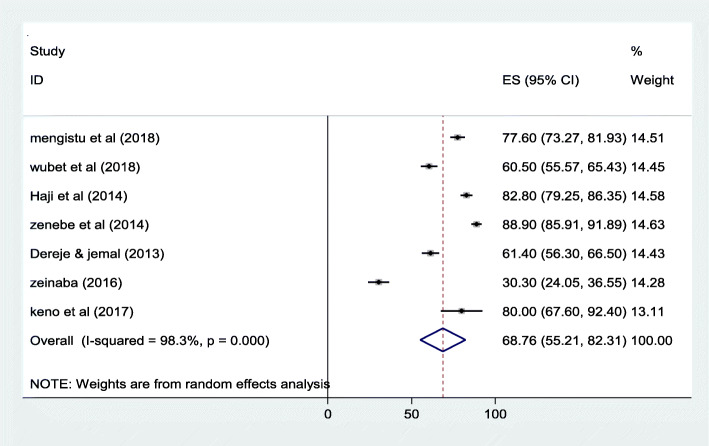
Forest plot of the pooled prevalence of iodine deficiency among pregnant women’s in Ethiopia, 2020

The pooled prevalence of iodine deficiency among pregnant women by using UIC level was 68.76% (95% CI: 55.21–82.31). High heterogeneity was observed across the included studies (I^2^ = 98.3%, *p* < 0.000). Hence, random effects model was used to calculate the pooled prevalence. See Fig. 2 below.

We tried to identify the source of heterogeneity. Possible factors such as year of publication, sample size, quality score, and region were examined using univariate Meta-regression. But none of the above factors were found to have a statistically significant effect (*P* > 0.05). See Table 2.

**Table 2:** Factors that might be related with heterogeneity of prevalence iodine deficiency among pregnant women in the current meta-analysis using univariate meta-regression

Variables	Coefficient	***P***-value
Sample size	0.0408	0.53
Quality score	16.116	0.38
Publication year	−1.384	0.761
**Region**		
Amahara	−10.977	0.56
Oromia (constant)	60.94	0.01

**Fig. 3:**
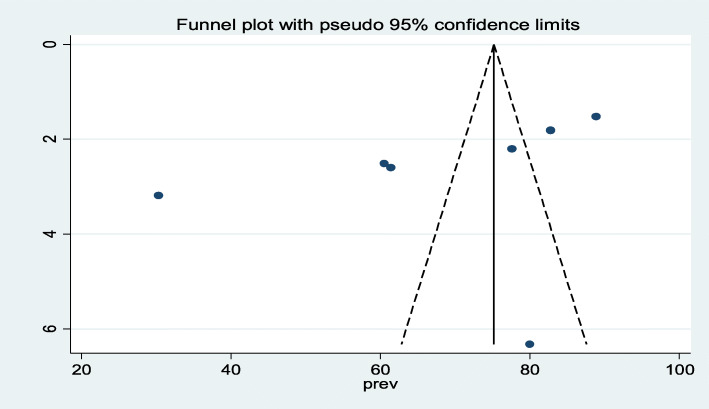
Funnel plot with 95% confidence limits of the pooled prevalence of iodine deficiency among pregnant women’s in Ethiopia, 2020

Presence of publication bias was evaluated by using Funnel plot and Egger’s test. The result of funnel plot showed that there was symmetrical distribution of articles (Fig. 3).

The result of Egger’s test for small-study effects showed that there was no statistically significant publication bias in estimating the prevalence of iodine deficiency (*P* = 0.149).

In addition, we conducted influential sensitivity (leave one out) analysis to see the effect of individual study on the observed heterogeneity. The results suggest that there is no single study that has high contribution for the heterogeneity. See Table 3.

**Table 3:** Showing the result of influential sensitivity analysis for prevalence of iodine deficiency among pregnant women in Ethiopia

Author	Year	Pooled prevalence	Confidence interval
Mengistu et al	2018	67.27	50.96–83.58
Wubet et al.	2018	70.16	55.07–85.24
Haji et al.	2014	66.38	49.80–82.96
Zenebe et al.	2014	65.31	50.63–79.99
Dereje & jemal	2013	70.00	54.84–85.17
Zeineba	2016	75.15	65.22–85.08
Keno et al	2017	67.06	52.35–81.80
Overall		68.76	55.21–82.31

Subgroup analysis was done using Region, where the studies were conducted and sample size. The highest prevalence of iodine deficiency was found in Oromia region 71.93% (95% CI: 54.87–88.99) followed by Amhara region 60.93%(95% CI: 57.39–64.48). The prevalence of iodine deficiency was higher in studies having a sample size of > 350, 77.58% (95% CI: 66.68–88.47) as compared to those having a sample size <= 350, 68.76% (95% CI: 55.21–82.31) (Table 4).

**Table 4:** Subgroup prevalence of iodine deficiency among pregnant women’s in Ethiopia (*n* = 7)

Variables	Characteristics	Included studies	Sample size	Prevalence
By Region	Amhara	2	728	60.93 (57.39–64.48)
	Oromia	5	1462	71.93 (54.87–88.99)
By sample size	> 350	4	1592	77.58 (66.68–88.47)
	< =350	3	598	68.76 (55.21–82.31)
Overall		7	2190	68.76 (55.21–82.31)

### Factors associated with iodine deficiency

#### Association between maternal education and iodine deficiency

**Fig. 4:**
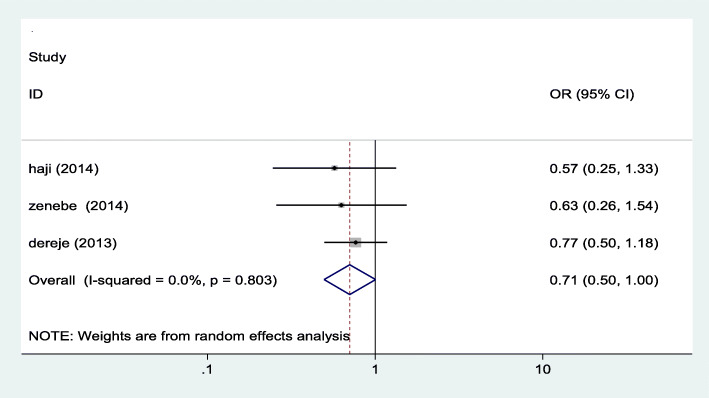
Forest plot showing pooled odds ratio of the association between maternal education and iodine deficiency in Ethiopia, 2020

In this meta-analysis, we examined association between maternal educational status and iodine deficiency using three studies [16–18]. The findings from these three studies revealed that there is no statistically significant association between maternal education and maternal iodine deficiency. See Fig. 4 below.

#### Association between iodized salt use and iodine deficiency

**Fig. 5:**
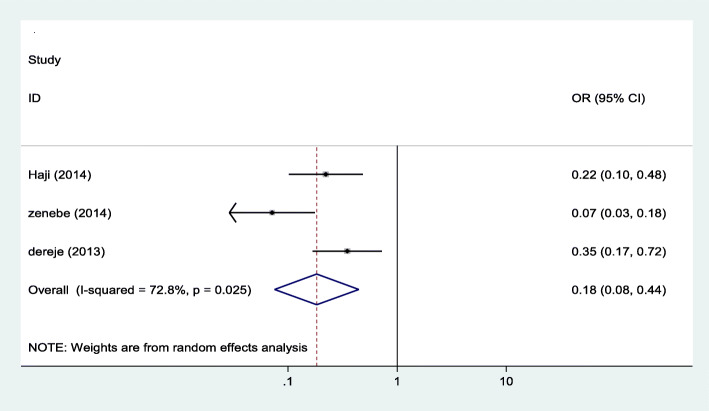
Forest plot showing pooled odds ratio of the association between use of iodized salt and iodine deficiency in Ethiopia, 2020

We examined the association between iodized salt use and iodine deficiency using three studies [16–18]. As shown on Fig. 5 below, the findings from these three studies revealed that using iodized salt reduces the odds of having iodine deficiency by 82% as compared to those who do not use iodized salt.

### Association between stages of pregnancy and iodine deficiency

Furthermore, in this review we have tried to see the association between stages of pregnancy and iodine deficiency. In this regard, iodine deficiency status was compared among different trimesters of pregnancy by using four studies [15–18].

**Fig. 6:**
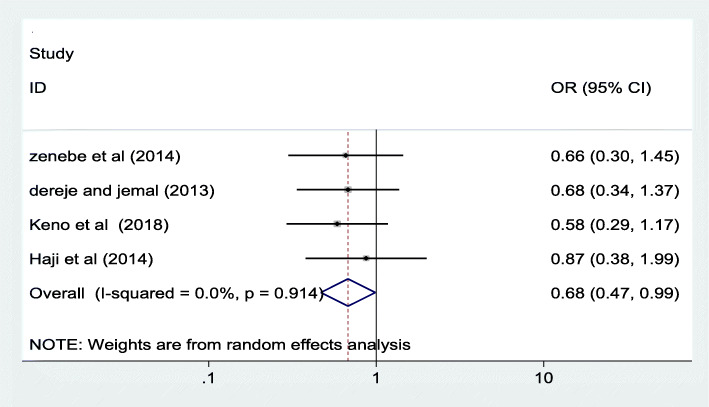
Forest plot showing pooled odds ratio of the difference in iodine deficiency between the first and second trimester in Ethiopia, 2020

**Fig. 7:**
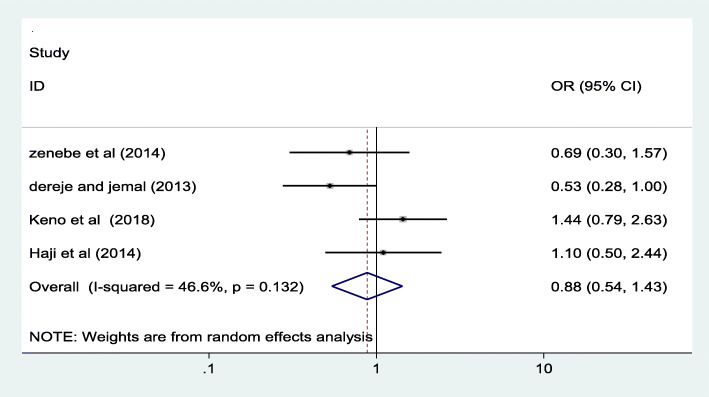
Forest plot showing pooled odds ratio of the difference in iodine deficiency between the first and third trimester in Ethiopia, 2020

**Fig. 8:**
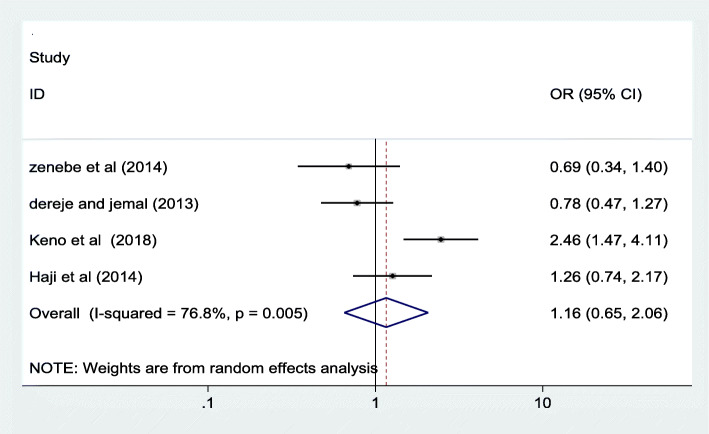
Forest plot showing pooled odds ratio of the difference in iodine deficiency between the second and third trimester in Ethiopia, 2020

I) **First trimester versus second trimester**: The odd of having iodine deficiency is reduced by 32% in the first trimester as compared to those in second trimester. See Fig. 6 below.
II) **‘First trimester versus third trimester’ and ‘second trimester versus third trimester’:** As shown in Fig. 7 below**,** the odds having iodine deficiency doesn’t show a statistically significant difference between the first and the third trimester. In addition, Fig. 8 below revealed that the likelihood of having iodine deficiency in the second trimester is not statistically different from the third trimester.

## Discussion

This systematic review and meta-analysis provide new evidence on the prevalence of iodine deficiency among pregnant women assessed with the use of biochemical methods employed in the included studies. The finding of this review revealed that the prevalence of iodine deficiency among pregnant women is 68.79% (95% CI: 56.21–82.31).

According to the recommendation by the technical consultation for prevention and control of IDD, a median UIC value between 150 μg/l and 249 μg/l during pregnancy defines a population that is not iodine-deficient [2]. The prevalence of iodine deficiency levels among pregnant women were also measured based on the median UIC value [21–26].

According to the world health organization, the prevalence of iodine deficiency among pregnant women found in this meta-analysis can be classified as a considerable public health problem [2]. This indicates that there is still a long journey to make significant progress in regions of Africa for reduction of iodine deficiency status among pregnant women. A study conducted on current global iodine status and progress over the last decade towards the elimination of iodine deficiency also classified Ethiopia among iodine-deficient countries [27]. A high prevalence of iodine deficiency in Ethiopia might be due to the mountainous topography of the country, which is prone to frequent erosion of the top layer of the soil for years leading to washing away of nutrients like iodine [21]. Furthermore, dietary habits that predispose to iodine deficiency like frequent of goitrogenic vegetables (such as cassava), cruciferous vegetables (cabbage and kale), and staple cereal crops (teff, wheat, lima beans, linseed, and sorghum) also play a role [22]. Besides, widespread use of iodine free (non iodized) table salt in Ethiopia could also be the main reason [23].

The finding of this study is comparable with that of a hospital-based longitudinal study conducted in Ghana which reported a 60.8% prevalence of iodine deficiency among pregnant women [28]. This finding is also supported by the results of a systematic review study conducted on insufficient iodine intake among pregnant women in different parts of the world; this review also reported that the median iodine intake was insufficient in 75% of the studies included [29].

On the other hand, the high prevalence of iodine deficiency among pregnant women in Ethiopia indicated by this review is contrary to the high coverage of iodized salt in the country. According to Ethiopian demographic health survey (EDHS) 2016, the coverage of iodized salt among households in Ethiopia was 89% [10]. Consistently, reports indicated that in 2017 85% of countries in Africa had achieved adequate iodine nutrition among the general population by using iodized salt. However, among the 11 countries with median urine iodine concentration (UIC) survey data of pregnant women, only four (South Africa, Tanzania, Sierra Leone, and Ghana) had sufficient iodine intake [27].

The discrepancy between iodine deficiency levels and the coverage of iodized salt might imply either a poor iodine content of the salt available in the households or poor utilization of the iodized salt during food preparation at the household level. In this regard, there is also low level of awareness on how to handle iodized salt from production to consumption. There is a culture of selling salt at outdoor market places such as local outdoor vendors and retails than supermarkets. Therefore, during this time there is exposed to sunlight. As iodine is a volatile substance, it evaporates when exposed to sunlight. This indicates the need for strict monitoring of iodized salt distribution and consumption at the household level.

Subgroup analysis using regions of the country showed that the prevalence of iodine deficiency among pregnant women in the Oromia region is 71.93% (54.87, 88.99). The prevalence in the Amhara region was slightly less than that of the Oromia region. This might be due to lesser number of studies that were analyzed from the Amhara region (only two studies) than those studied in the Oromia (five studies). Regardless of the difference in number of studies conducted in the two regions, the findings from both regions have a comparable level of iodine deficiency, which is still a public health problem [21]. Almost all of the studies included in this meta-analysis were conducted in the central and the northern parts of Ethiopia, which is mostly known by its widespread agriculture (cultivating crops and herding animals) [23]. As a result, crop cultivation and overgrazing in the specified areas might make the land prone to erosion leading to depletion of micronutrients including iodine.

In this review, we also tried to examine the association of some factors with iodine deficiency among pregnant women. The association between utilization of iodized salt and iodine deficiency among pregnant women was computed using three primary studies from the total of 7 articles included in this review. The finding revealed that the likelihood of iodine deficiency is reduced by 82% by using iodized salt. This finding in line with other studies conducted in Ethiopia that reported poor iodized salt utilization as the main predisposing factor to a high prevalence of iodine deficiency [24–26]. A similar finding was reported from a systematic review and meta-analyses study conducted by WHO [30]. Hence, knowing the fact that salt is the only food item used for iodization in Ethiopia, it is evident that poor utilization of iodized salt is the main contributing factor for the high prevalence of iodine deficiency among pregnant women, and this is why the use of iodized salt is recommended as an effective strategy for reducing iodine deficiency [27].

Even though some of the individual studies included in this review has reported as there is a significant association between maternal educational status and iodine deficiency, the pooled odds ratio of this review revealed no significant association [0.71 (95% CI: ((0.50, 1.00)]. Similarly, there is no significant association between iodine deficiency and the stages of pregnancy. But statistically significant marginal association was observed between the first trimester and iodine deficiency as compared to those in second trimester. The association of the stage of pregnancy with iodine deficiency was also insignificant in almost all of the included studies [16–18] and similar findings were reported from other studies conducted elsewhere [31–33].

### Limitations of the study

One of the limitations of this study was only articles written in English were considered in this review, which may result in the exclusion of other articles. This Meta-analysis represented studies reported from only two regions of the country, which may reflect as under-representation since few articles were found and included. All the studies included in this review were cross sectional studies, thus results generated in this study should be interpreted with caution since the quality of evidence produced from cross sectional studies is relatively low.

All included studies reported a single UIC measurement, which might not clearly show iodine deficiency at an individual level, rather at a population level. There are also variations from one study to another study in different aspects of urine iodine measurement like urine storage, transportation, time of measurement by each study. Such variations might explain the high heterogeneity of estimates observed in the current study.

## Conclusions

Iodine deficiency among pregnant women remains to be a major public health problem in many parts of Ethiopia. Iodized salt utilization at the household level was significantly associated with iodine deficiency among pregnant women. Although the iodized salt coverage in Ethiopia is reportedly high (89.2%), the prevalence of IDD among pregnant women remained significantly high. This finding implies that the prevalence of iodine deficiency among pregnant women in Ethiopia still needs due consideration and efforts has to be made by the government to increase accessibility and utilization of iodized salt.

Hence, intervention aiming at preventing iodine deficiency in the country including the use of iodized salt as the only primary option for iodine source at the household level might need re-consideration. Particularly, other options like processed foods that are being practiced in some countries that might boost the effect of iodized salt particularly among pregnant women needs consideration.

## Supplementary Information

**Additional file 1: Supplemental file 1.** Summary of search results for the PubMed, Google Scholar and other databases.

**Additional file 2: Supplemental file 2.** Quality score of each study.

**Additional file 3: Supplemental file 3.** PRISMA (Preferred Reporting Items for Systematic Reviews and Meta-Analyses) checklist.

## Data Availability

Data will be available upon reasonable request of the corresponding author.

## Abbreviations

AOR: Adjusted odds ratio
CI: Confidence interval
EDHS: Ethiopian demographic health survey
GC: Gregorian calendar
ID: Iodine deficiency
IDD: Iodine deficiency disorder
IQ: Intelligence quotient
PRISMA: Preferred Reporting Items for Systematic Reviews and Meta-Analyses
RNI: Recommended nutrient intake
UI: Urine iodine
UIC: Urine iodine concentration
UNICEF: United Nations international children’s emergency fund
WHO: World health organization
