# Supplementary material for: Systematic review and meta-analysis of iodine deficiency and its associated factors among pregnant women in Ethiopia
Source: BMC Pregnancy Childbirth. 2021 Feb 4;21:106. doi: 10.1186/s12884-021-03584-0 (PMC7863485; doi:10.1186/s12884-021-03584-0)
Supplement: Supplementary file 1 — Additional file 1: Supplemental file 1. Summary of search results for the PubMed, Google Scholar and other databases. [file 12884_2021_3584_MOESM1_ESM.docx]

Supplemental Table 1

| **Databases** | **Searching terms** | **Number of studies** |
| --- | --- | --- |
| Google scholar | “prevalence” or “magnitude” and “iodine” and “Deficiency” or “level” and “pregnancy” or “pregnant” or “mothers” and “Ethiopia” | 794 |
| PubMed | Search ((("iodine"[MeSH Terms] OR "iodides"[MeSH Terms] OR iodine[Text Word])) AND ("deficiency"[Subheading] OR deficiency[Text Word])) AND ("pregnancy"[MeSH Terms] OR pregnancy[Text Word])) AND (("Ethiopia"[MeSH Terms] OR Ethiopia[Text Word]) Filters: published in the last 10 years | 268 |
| From other databases* |  | 54 |
| Total retrieved articles |  | 1116 |
| Full text papers appropriate to our review |  | 7 |

* Web of science, CINAHL and global health
