# Supplementary material for: Systematic review and meta-analysis of iodine deficiency and its associated factors among pregnant women in Ethiopia
Source: BMC Pregnancy Childbirth. 2021 Feb 4;21:106. doi: 10.1186/s12884-021-03584-0 (PMC7863485; doi:10.1186/s12884-021-03584-0)
Supplement: Supplementary file 2 — Additional file 2: Supplemental file 2. Quality score of each study. [file 12884_2021_3584_MOESM2_ESM.docx]

| **Table Showing quality score of each study using Newcastle-Ottawa quality assessment tool adapted for cross-sectional studies**. | | | | | | | |
| --- | --- | --- | --- | --- | --- | --- | --- |
| **Sample selection (maximum 5 stars)** | Mengistu et al. | Wubet et al. | Haji et al. | Zenebe et al. | Dereje & Jemal | Zeinaba | Keno et al |
| 1. **Representativeness of the sample**: Truly representative of the average in the target population. * (all subjects or random sampling), b) Somewhat representative of the average in the target population. * (non-random sampling), c) Selected group of users and d) No description of the sampling strategy | a* | c | a* | c | c | c | c |
| 2. **Sample size**: a) **Justified and satisfactory.** * And b) Not justified. | * | * | * | * | * | * | b |
| 3. **Non-respondents**: a) Comparability between respondents and non-respondents characteristics is established, and the ***response rate is satisfactory.*** *, b) The response rate is unsatisfactory, or the comparability between respondents and non-respondents is unsatisfactory and c) No description of the response rate or the characteristics of the responders and the non-responders. | * | * | * | * | c | c | c |
| 4) **Ascertainment of the exposure (risk factor)**: a) Validated measurement tool. **, b) Non-validated measurement tool, but the tool is available or described. *, c) No description of the measurement tool. | a** | a** | a** | a** | a** | a** | a** |
| **Comparability: (Maximum 2 stars)** |  |  |  |  |  |  |  |
| 1) The subjects in different outcome groups are comparable, based on the study design or analysis. Confounding factors are controlled. a) The study controls for the most important factor (select one). * b) The study control for any additional factor. * | b* | b* | b* | b* | b* | b* | a* |
| **Outcome: (Maximum 3 stars)** |  |  |  |  |  |  |  |
| 1) Assessment of the outcome: a) Independent blind assessment. **, b) Record linkage. ** c) Self report. * and d) No description. | b** | b** | a** | a** | a** | a** | a** |
| 2) Statistical test: a) The statistical test used to analyze the data is clearly described and appropriate, and the measurement of the association is presented, including confidence intervals and the probability level (p value). * and b) The statistical test is not appropriate, not described or incomplete. | * | * | * | * | * | * | * |
| **Overall quality score (máximum of ten stars)** | 10 | 9 | 10 | 9 | 9 | 9 | 8 |
